# A Precise Pulmonary Airway Tree Segmentation Method Using Quasi-Spherical Region Constraint and Tracheal Wall Gap Sealing

**DOI:** 10.3390/s24165104

**Published:** 2024-08-06

**Authors:** Zhanming Hu, Tonglong Ren, Meirong Ren, Wentao Cui, Enqing Dong, Peng Xue

**Affiliations:** School of Mechanical, Electrical and Information Engineering, Shandong University, Weihai 264209, China

**Keywords:** pulmonary airway tree, segmentation, wavefront propagation, leakage, EXACT09

## Abstract

Accurate segmentation of the pulmonary airway tree is crucial for diagnosing lung diseases. To tackle the issues of low segmentation accuracy and frequent leaks in existing methods, this paper proposes a precise segmentation method using quasi-spherical region-constrained wavefront propagation with tracheal wall gap sealing. Based on the characteristic that the surface formed by seed points approximates the airway cross-section, the width of the unsegmented airway is calculated, determining the initial quasi-spherical constraint region. Using the wavefront propagation method, seed points are continuously propagated and segmented along the tracheal wall within the quasi-spherical constraint region, thus overcoming the need to determine complex segmentation directions. To seal tracheal wall gaps, a morphological closing operation is utilized to extract the characteristics of small holes and locate low-brightness tracheal wall gaps. By filling the CT values at these gaps, the method seals the tracheal wall gaps. Extensive experiments on the EXACT09 dataset demonstrate that our algorithm ranks third in segmentation completeness. Moreover, its performance in preventing airway leaks is significantly better than the top-two algorithms, effectively preventing large-scale leak-induced spread.

## 1. Introduction

In recent years, the incidence of pulmonary diseases, including infections, diffuse diseases, tracheal and bronchial diseases, and lung cancer, has rapidly increased. Thus, precise diagnosis of these conditions has become crucial. Accurate segmentation of the pulmonary airway tree in clinical practice helps locate and identify diseased lung areas, aiding doctors in formulating personalized treatment plans. For instance, precise segmentation of the pulmonary airway tree allows doctors to assess structural changes, such as tracheal wall thickening, airway narrowing, and areas of airflow restriction. These assessments are crucial for determining the severity and classification of COPD [1,2]. Additionally, three-dimensional visualization of the pulmonary airway tree is essential for virtual bronchoscopy. Segmenting the pulmonary airway tree enables three-dimensional visualization of the airway structure, significantly improving examination accuracy and safety [3,4]. Moreover, precise segmentation of the pulmonary airway tree can serve as a basis for segmenting other lung structures, aiding in accurate segmentation of other lung anatomical structures [5,6,7,8].

**Figure 1:**
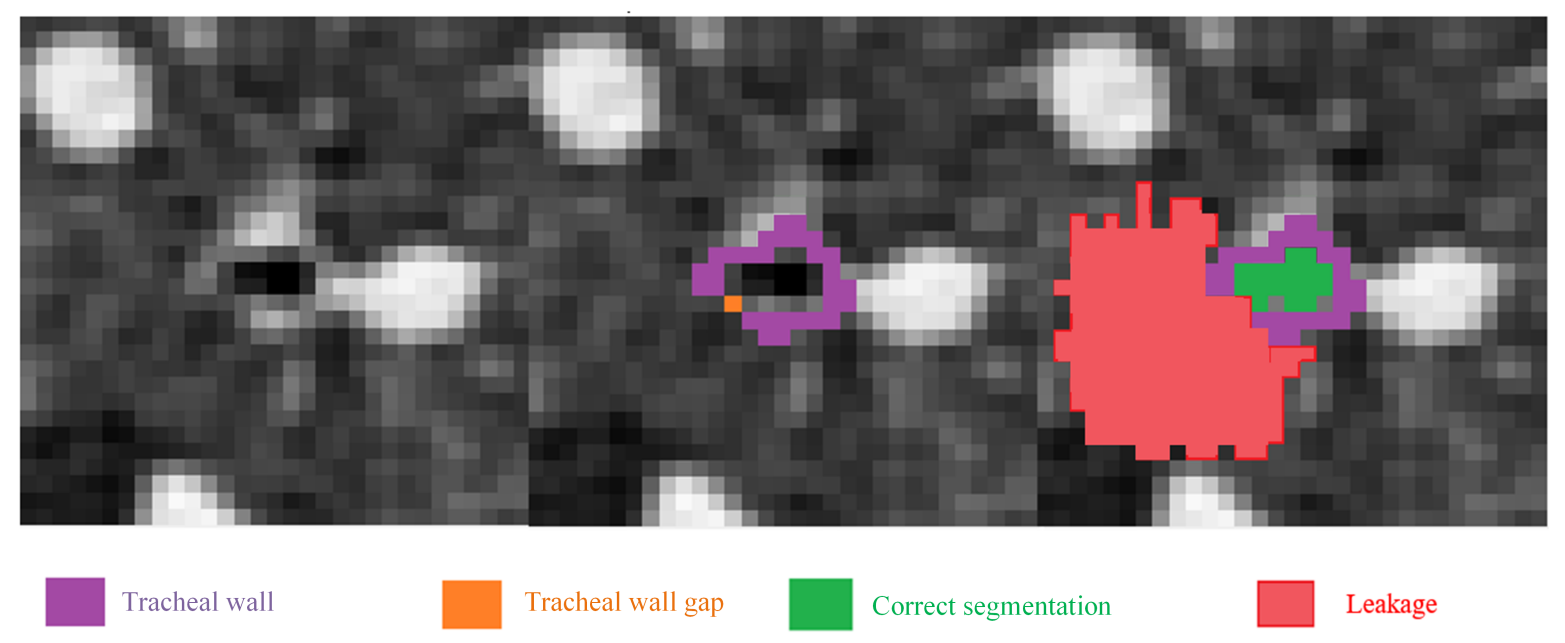
When there is a gap in the tracheal wall, the segmentation direction will be guided to the outside of the trachea, which is the red part, causing incorrect segmentation. If the tracheal wall is not promptly sealed and leaks are not cleared, incorrect segmentation may spread throughout the entire lung parenchyma.

In pulmonary airway tree segmentation, common issues include significant variations in tissue shape and size, uneven grayscale between the target and background, low and unstable contrast, and unclear and incomplete edges. Additionally, two specific challenges are the low completion of small airway segmentation and high susceptibility to leakage. As shown in Figure 1, leakage can cause non-airway tissues to be incorrectly segmented as airways, directly reducing segmentation accuracy. For instance, lung parenchyma or blood vessels, which are non-airway tissues, may be mistaken for airways, increasing the error area in the segmentation results. Inaccurate segmentation results due to leakage may lead doctors to make diagnostic and treatment decisions based on incorrect airway tree structures. Conversely, some algorithms—to effectively control leakage during segmentation—fail to extract the precise structures of the pulmonary airway tree, resulting in low segmentation completeness. Thus, balancing complete and precise segmentation of small airways with leakage control is challenging [9].

Recently, with advancements in computer hardware, deep learning-based methods for pulmonary airway tree segmentation have progressed significantly. Wu et al. [10] introduced a framework featuring dual training phases and a 3D contextual transformer module for segmenting the entire pulmonary airway and small airway branches. This method has two training stages and uses a modified 3D U-Net network, integrating a 3D contextual transformer block to capture contextual and long-range information effectively. Lyu et al. [11] introduced a novel dual encoder network designed for precise pulmonary airway segmentation. This method combines convolutional neural networks (CNNs) and Transformer networks, capturing local and global features, as well as the unique tree structure and fine details of terminal bronchioles effectively. Guo et al. [12] developed a multi-information fusion convolutional neural network (Mif-CNN) and a CNN-based region-growing method for segmenting the entire airway and small branches. By integrating boundary and positional information, the Mif-CNN leverages additional contextual knowledge and features, focusing on acquiring precise airway branches using the CNN-based region-growing method.

However, the aforementioned deep learning-based methods necessitate annotations of the training set by specialized physicians. Additionally, deep learning-based pulmonary airway tree segmentation methods are prone to overfitting with limited medical image datasets, lacking generalization capability. Therefore, we have refocused on traditional methods for pulmonary airway tree segmentation. Traditional pulmonary airway segmentation algorithms can be classified into three main types: knowledge-based segmentation, region-growing/wavefront propagation, and centerline extraction [13]. Knowledge-based segmentation methods are often used for leakage determination as an auxiliary segmentation strategy but are less commonly used due to high computational demands [14]. Centerline extraction methods refine segmentation to derive the centerline and then use morphological processing to stitch broken airways together. This method requires post-processing segmentation results, has high computational demands, and exhibits low robustness [15,16]. Region growing methods combine edge detection and gradient information to precisely locate target boundaries [17].

The wavefront propagation method’s segmentation concept is similar to region growing and is often categorized as such. However, the wavefront propagation method introduces the concept of a wavefront, likening the iterative segmentation process to wave propagation radiating in all directions from the wave source. Therefore, the wavefront propagation method can flexibly handle topological changes during segmentation, such as branching and merging, making it well suited for targets with complex branching structures like the airway tree and ensuring segmentation results’ completeness and continuity. Additionally, compared to traditional region-growing algorithms, the wavefront propagation method simplifies obtaining the set of points to be segmented using morphological dilation, eliminating the need for segmentation direction calculations and significantly reducing computation time and hardware demands. However, omitting segmentation direction calculation simplifies the iterative process of the wavefront propagation method but introduces a clear disadvantage. Kumar et al. [18] noted that the direction in the wavefront propagation method is difficult to control during point collection before threshold determination, easily causing directional errors and leading to leakage. Atilla et al. [19] also employed a method combining region growing and morphological operations, achieving some segmentation accuracy but causing significant leakage.

To overcome this challenge, this paper uses a constrained region model to limit leakage and compensate for wavefront propagation algorithm shortcomings. The constrained region model encloses segmentation results, enabling the timely control of leakage caused by erroneous segmentation and containing the leakage within the region. The constrained region model can identify the number of airway branches in the current segmentation result, adjust the direction for the next segmentation, and automatically change the model size to match the airway width. Therefore, this paper uses the wavefront propagation method with the constrained region model as an auxiliary for segmentation. The constrained region model can limit leakage within a certain area, thereby reducing leakage. However, existing constrained models come in various shapes, and inappropriate models are difficult to match with airway shapes, resulting in increased segmentation time. To address these shortcomings, this paper proposes a quasi-spherical constrained region, within which segmentation is performed that limits leakage diffusion.

To address leakage within the constrained region, this paper proposes a method for tracheal wall occlusion to maximally control leakage. When there is a gap in the tracheal wall, dilation and erosion operations locate and fill gaps without altering the tracheal wall structure. In our proposed method, leakage determination is more closely related to region morphology and airway width, making the constrained region method more significant in leakage determination. The quasi-spherical constrained region method matches the iterative approach of the wavefront propagation method and provides more stable region edges in the three-dimensional structure. In summary, the main contributions of this paper are as follows:

- This paper proposes a quasi-spherical region constraint method. By approximating the airway cross-sectional area from the voxel-formed surface area, an approximate spherical constraint region is determined based on the width of the small airway segment for segmentation. Compared to common direction-assisted constraint regions, the direction-independent quasi-spherical constraint is better suited for the complex and intricate branches of the pulmonary airway.
- A tracheal wall gap sealing algorithm is proposed. By determining leakage at the region edges and adaptively adjusting the threshold, it effectively limits large-scale diffusion after leakage occurs. When erroneous segmentation occurs, leakage is controlled within the quasi-spherical region. The tracheal wall gaps are located through morphological closing operations and promptly occluded.

After validation with EXACT09 [20] standards and comparison with 21 other publicly available high-performance algorithms, the proposed algorithm ranked third in four parameters indicating segmentation completeness. However, it significantly outperformed the top-two algorithms in leakage control, demonstrating stronger overall performance and robustness. Compared to the remaining three parameters indicating segmentation error rates, the proposed algorithm can occlude most small tracheal wall gaps, significantly limiting under-segmentation and showing notable effectiveness in small airways.

## 2. Related Work

### 2.1. Pulmonary Airway Tree Segmentation Based on Wavefront Propagation Algorithm

Aykac et al. [21] categorized common airway segmentation methods into four additional types: Anatomical Knowledge-Based, Region-Growing, Fuzzy Logic (fuzzy connectivity), and Mathematical Morphology. After years of continuous research, scholars have found that single algorithms are insufficient to address the many issues in airway tree segmentation. Thus, research and improvements in algorithms often focus on technical crossover or fusion. Duan et al. [22] combined a two-pass region-growing algorithm with gray-level morphological reconstruction and leakage elimination, proposing a leakage cleaning method that detects and removes leakage, refines the airway tree, and reduces the false positive rate after airway segmentation. Graham et al. [23] combined airway tree structure with region growing using local image features to guide airway tree segmentation. Frimmel et al. [24] proposed a hybrid method of morphological dilation involving skeleton centerline extraction and region growing. However, the skeleton centerline extraction method they used is prone to deviation in low-dose CT images. Anna et al. [25] employed a two-step segmentation method using morphological erosion and dilation to detect possible locations of distal airways. Inspired by the above methods, this paper builds upon and improves the wavefront propagation method by incorporating a constrained region model for segmentation.

### 2.2. Region Constrained Model

Tschirren et al. [26] proposed a method based on the region of interest partitioning to prevent large-scale leakage into the lung parenchyma during segmentation, thereby improving algorithm efficiency. Region-constrained models can be categorized into direction-assisted region-constrained methods and non-direction-assisted region-constrained methods based on the presence or absence of a central axis. For direction-assisted region constrained methods, the anatomical structure of the airway tree in non-branching regions is similar to a pipeline, thereby approximating a cylinder. Therefore, cylindrical region constraints are widely used in many airway tree segmentation algorithms based on the wavefront propagation method. Most methods predefine a 3D cylindrical template to match the tubular structure, and during segmentation, the segmentation direction needs to approximate the direction of the cylindrical central axis. Pechin et al. [27] proposed an airway tree segmentation method based on pulmonary vessel guidance utilizing the characteristic that vessels and airway structures are tubular and accompany each other in the same direction to guide segmentation, effectively identifying and suppressing leakage. However, this method requires defining the vessel direction and locating the accompanying airway segments before segmentation, increasing algorithm complexity and computation time. Lee et al. [28] applied and improved this constrained method in their adaptive cylindrical constrained region-growing algorithm. Lidayová et al. [29] detected the approximate airway centerline tree and then applied the obtained intensity and distance information to constrain onion–kernel region-growing segmentation, preventing leakage into thin-walled tissues. Graham et al. [23] first applied region growing for conservative segmentation of the trachea and main branches and then scanned and located all airway cross-sections by applying elliptical decision functions on three 2D planes (transverse, coronal, and sagittal).

Another method is the box region-constrained model proposed by Feuerstein et al. [30]. The core idea of box region constraint remains direction-assisted region constraint, similarly calculating direction and box size based on previous segmentation results and setting regional constraints according to branching conditions. This method leverages the change in the number of skeleton center points by calculating the positions of the center points before and after branching to obtain a relatively precise direction. Additionally, the separation of branching and non-branching processes is a significant feature of this algorithm, effectively avoiding the impact of abrupt increases and decreases in airway width during branching on leakage detection in non-branching states. However, in practical operations, the added complexity of skeleton centerline extraction and branching direction calculation increases segmentation time, making it unsuitable for the iterative segmentation of large datasets in airway tree segmentation. To overcome the challenges posed by the complex branching directions of the airway tree and avoid direction errors causing leakage, researchers have proposed direction-independent constrained models, i.e., non-direction-assisted region-constrained methods.

The direction-independent region constrained method is rarely used in the related literature because its constrained regions are difficult to match the airway shape during segmentation. However, due to the omni-directional nature of the wavefront propagation method, which does not require direction calculation, it is better suited to the direction-independent region constrained method. Additionally, the direction-independent region-constrained method does not require direction calculation, resulting in lower computational complexity. James et al. [8] adopted the direction-independent region-constrained method during the use of the wavefront propagation method, setting a spherical region constraint with a diameter slightly larger than the tube segment diameter. However, in leakage determination, the algorithm calculates the segmentation direction and the angle with the reference direction, abandoning the advantage of the wavefront propagation method’s direction-independent calculation, increasing computational complexity and making it less effective than region-growing-based algorithms. The direction-independent region constrained method better matches the wavefront propagation method, which does not require direction calculation. However, existing region-constrained methods have flaws, necessitating the search for new region-constrained methods suitable for the wavefront propagation method.

**Figure 2:**
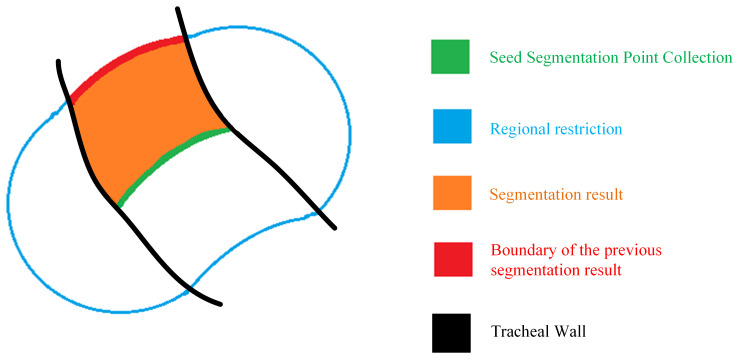
In the pulmonary airway, the constrained region encloses the area to be segmented. The segmentation iterates continuously toward the ends of the airway. With each iteration, the size of the quasi-spherical constrained region changes, and its position moves along with the area to be segmented towards the airway terminal.

The proposed quasi-spherical region constraint model calculates the radius based on airway width and marks the region through a single-time dilation from the seed point set. Subtracting the result of the radius decay from the marked region yields the boundary of this region, as shown in the simplified process in Figure 2. Except for the first round where the segmentation seed point is a single point, the seed point set is the intersecting surface of the segmentation result within the previous region and the region boundary. Therefore, the constrained region set by this method is not a regular sphere; we hence the name quasi-spherical region constraint.

### 2.3. Balancing Small Airway Segmentation and Leakage

Balancing the segmentation of small airways and controlling leakage has always been a significant challenge in airway tree segmentation. Tschirren et al. [26] proposed a method based on the segmentation of regions of interest to prevent large-scale leakage into the lung parenchyma, thereby improving algorithm efficiency. However, experimental results show that this method cannot adequately detect leakage in distal airways, resulting in suboptimal segmentation performance. For the determination of small airways, Bartz et al. [31] used histogram theoretical methods to quantitatively describe chest CT data and established the following rules: CT values below −950 HU are definitely airway regions, values above −775 HU are definitely non-airway regions, and values between these thresholds are uncertain regions. Small airways, affected by partial volume effects, often blur the boundary CT values between the airway and the tracheal wall, leading to their classification as uncertain regions. This results in existing airway tree segmentation methods easily causing leakage in these uncertain regions. To prevent leakage, researchers have proposed numerous airway enhancement methods, with grayscale morphological reconstruction being the most commonly used [32]. Currently, many auxiliary rules based on encoded imaging and anatomical knowledge help determine whether uncertain regions are small airways, such as (1) local contrast between the lumen and wall [33]; (2) the airway centerline and morphological structure [15]; and (3) the similarity between the airway and the parallel pulmonary artery [9]. This specialized combination of enhancement and rules can improve extraction accuracy but also increases time costs. Regardless of the additional strategies used, balancing complete segmentation and leak-free segmentation remains a complex task. Many true airway branches are discarded along with excessively segmented lung tissue to reduce leakage. The loss of these branches leads to the complete loss of their underlying anatomical structures, even though these airways are still visible on a CT image.

Under these rules, erroneous segmentation leakage could be effectively avoided in their study. However, in the meantime, the segmentation quality of small airways was degraded. As a result, the intention of the maximum segmentation of airways was not improved. Therefore, a method is needed to control leakage within the constrained region model when small airway segmentation leakage occurs. In airway tree segmentation, the CT appearance of the lung parenchyma outside the airway is similar to that of the airway, leading to the continuous expansion of erroneous segmentation. This has led to two approaches for handling leakage: (1) preventing erroneous expansion by quickly erasing the starting point of erroneous segmentation, resulting in numerous real-time detection methods, which are difficult to implement and have poor adaptability; and (2) utilizing the continuous expansion of errors, setting a certain range to detect leakage (erroneous segmentation), then adjusting parameters and re-segmenting. The larger the detection range, the more obvious the erroneous segmentation, and the easier the leakage detection, but repeated large-scale segmentation leads to longer algorithm run times and lower practical application value. Each approach has its own characteristics, so a combined method should be used to block erroneous segmentation within the established constraint region and seal the leakage.

## 3. Methods

This paper integrates quasi-spherical region constraints with the wavefront propagation method to enhance the fine segmentation of small pulmonary airway trees. By considering the local segmentation environment and the width of the targeted small airway segment, an approximately spherical constraint region is established. Within this region, leakage is detected, and parameters are adjusted accordingly. Morphological methods are utilized to identify low-brightness tracheal wall gaps within the constraint region, where the CT values are filled to seal these gaps. This enhancement improves the boundary delineation of the tracheal wall during segmentation. Ultimately, this approach addresses common challenges such as large-scale leakage and low segmentation completeness in airway tree segmentation.

### 3.1. Quasi-Spherical Region Constraint

Region-based segmentation is a commonly utilized auxiliary method in airway tree segmentation research. The shape and size of the segmentation results at the edge of the previous region can be leveraged for the subsequent segmentation step. However, the variable shapes of cylindrical and rectangular region edges often compromise algorithm stability. Therefore, this paper adopts a region constraint method with purely curved edges. Since the region of interest is obtained by a single dilation of a set of seed points with an irregular three-dimensional shape using a spherical operator, this region forms an irregular sphere, hence termed the quasi-spherical region constraint model. During the segmentation process, the seed point set at a particular stage is a small surface formed by the intersection of the previous stage’s segmentation results with the region edge. At a particular stage, different regions may form multiple small surfaces, which are all stored in the same matrix as binary images. Using a connected domain detection function, many unconnected small surfaces can be located and marked, allowing each small surface to be extracted separately and segmented independently at this stage.

Before commencing segmentation using the small surfaces as seed point sets, quasi-spherical region initialization is required. This includes the three-dimensional construction of the quasi-spherical constraint region, the calculation of initial parameters within the region, image cropping, and pre-processing. The specific steps are as follows: (1) Detect connected domains and mark them to determine the positions of multiple seed point sets and form a stack; (2) extract single connected seed point sets from the stack sequentially; (3) calculate the airway cross-sectional area for the seed point set and compute the region radius (*R*) and initial threshold; (4) crop the small image segment to be segmented (optimization process); (5) selective preprocessing; (6) region constraint and boundary generation. Since the seed point set surface is approximately equivalent to the airway cross-section, the total number of voxel points on this surface is used to replace the airway cross-sectional area. The initial region radius (*R*) is calculated as shown in Equation (1):

(1) $$R=3\times ceil\left[\frac{\sum_{x=1}^{\max}\sum_{y=1}^{\max}\sum_{z=1}^{\max}f_{\mathrm{vary}}\left(K\right)\left(x,y,z\right)}{100}\right]+5$$

where *K* is the *K*th seed point set. $f_{area}\left(x,y,z\right)$ is the three-dimensional binary image function of the current small surface, and ceil represents the ceiling operation. The initial threshold for segmentation at this stage is also related to this small surface. The initial range of variation for threshold setting is determined by the range of CT values represented by the position of the small surface in the chest CT image. Assuming the original image *A* is $f_{A}\left(x,y,z\right)$, with a range of $f_{A}\left(x,y,z\right)\in \mathrm{CT}$, the variation in CT values of voxel points on the seed point surface is given by Equation (2):

(2) $$f_{\mathrm{vary}}\left(K\right)\left(x,y,z\right)=f_{\mathrm{area}}\left(K\right)\left(x,y,z\right)\cdot f_{A}\left(x,y,z\right)$$

Therefore, the initial threshold is set as

(3) $$Seed\left(K\right)=\min\left[f_{\mathrm{vary}}\left(K\right)\left(x,y,z\right)\right]$$

(4) $$Threshold\left(K\right)=\max\left[f_{\mathrm{vary}}\left(K\right)\left(x,y,z\right)\right]-\min\left[f_{\mathrm{vary}}\left(K\right)\left(x,y,z\right)\right]+1$$

where the minimum CT value is denoted as Seed, and the threshold is denoted as *Threshold*. To reduce computational demand and segmentation time, the 3D image data involved in iterations should not be too large. Therefore, the 3D matrix involved in the iterations is segmented to optimize the algorithm, retaining only small 3D matrices relevant to the algorithm’s region constraints for processing. The specific steps are as follows: (1) Identify the seed point surface boundaries, i.e., the value ranges for the three coordinate axes; (2) expand the boundaries by R voxel distances, ensuring not to exceed the CT image boundaries; (3) $T\left(K\right)=f_{T}\left(K\right)\left(x,y,z\right)=f_{A}\left(x_{\min}∼x_{\max},y_{\min}∼y_{\max},z_{\min}∼z_{\max}\right)$ is the image after cutting the original image; (4) $P_{\mathrm{area}0}\left(K\right)=f_{\mathrm{area}}\left(K\right)\left(x_{\min}∼x_{\max},y_{\min}∼y_{\max},z_{\min}∼z_{\max}\right)$ is the seed point matrix to be segmented. Next, the tracheal wall needs to undergo enhancement pre-processing. The partial derivatives along each coordinate axis are calculated to obtain the local maxima of CT value changes, and after computational superposition, the local peak matrix is obtained. Through refinement processing, a thinner tracheal wall matrix to be enhanced is derived. By superimposing it on the original data, data enhancement is achieved. Preprocessing can limit leaks in medium-width airways and also restrict leaks that are prone to occur at the trachea. On this small image block, a three-dimensional structure resembling a sphere is generated, and the boundary of the region is extracted. Outside the region, the image is assigned the minimum CT value, preventing segmentation beyond the region and effectively limiting its effect. The functions to generate the boundary of the sphere-like region and the image restricted to this region are as follows:

(5) $$T_{\mathrm{edgy}}=f_{\mathrm{edge}}\left(x,y,z\right)=\mathrm{Imdilate}\left[P_{\mathrm{area}0}\right.,Sphere\left.\left(R\right)\right]-\mathrm{Imdilate}\left[P_{\mathrm{area}0}\right.,Sphere\left.\left(R-1\right)\right]$$

(6) $$T_{\mathrm{region}}=f_{\mathrm{region}}\left(x,y,z\right)=\mathrm{Imdilate}\left[P_{\mathrm{area}0}\right.,Sphere\left.\left(R\right)\right]\cdot f_{T}\left(x,y,z\right)-2048\cdot {\left\{\mathrm{Imdilate}\left[P_{\mathrm{area}0}\right.,Sphere\left.\left(R\right)\right]\right\}}^{c}$$

where Tedgy and Tregion represent the boundary and restriction images of the spherical region, respectively. Sphere(R) is the spherical operator with a radius of *R*. Imdilate represents morphological dilation operation. $P_{\mathrm{area}0}$ represents the current seed point matrix to be segmented. $f_{T}\left(x,y,z\right)$ is the function of small block CT images involved in the iteration. The value -2048HU is the minimum value of CT image. *c* represents the inverse operation of binary image, i.e., conversion between values 0 and 1.

### 3.2. Region-Constrained Wavefront Propagation

The wavefront propagation method is initiated from a set of seed points for segmentation. It calculates the similarity between neighboring voxels and the seed point set in terms of grayscale (represented as CT values in medical CT images), texture, etc. Based on predefined rules (such as thresholding), it determines whether adjacent voxels should be added to the segmented region. Through iterative processes, the segmentation results are progressively accumulated, eventually traversing each voxel to achieve segmentation of the entire image. Generally, the important steps of the wavefront propagation method are as follows:

1. Seed Point Selection
  Seed point selection is divided into two types: algorithmic automatic selection and interactive selection. For multi-target images, one seed point (or set of seed points) needs to be selected in each target area. In pulmonary airway tree segmentation, only one region needs to be segmented; thus, a single seed point suffices to initiate segmentation.
2. Voxel Selection or Rejection
  Thresholding is one of the most commonly used determination methods in pulmonary airway tree segmentation. Thresholding can be classified into static or dynamic based on whether the threshold is set as a constant or variable. For simple images, static thresholding can complete the segmentation process. However, for complex images, additional information such as fuzzy connectivity and texture feature recognition needs to be incorporated on top of dynamic thresholding for selection or rejection determination. In pulmonary airway tree segmentation, a complex and precise process for leakage (false segmentation) detection needs to be established based on dynamic thresholding.

**Figure 3:**
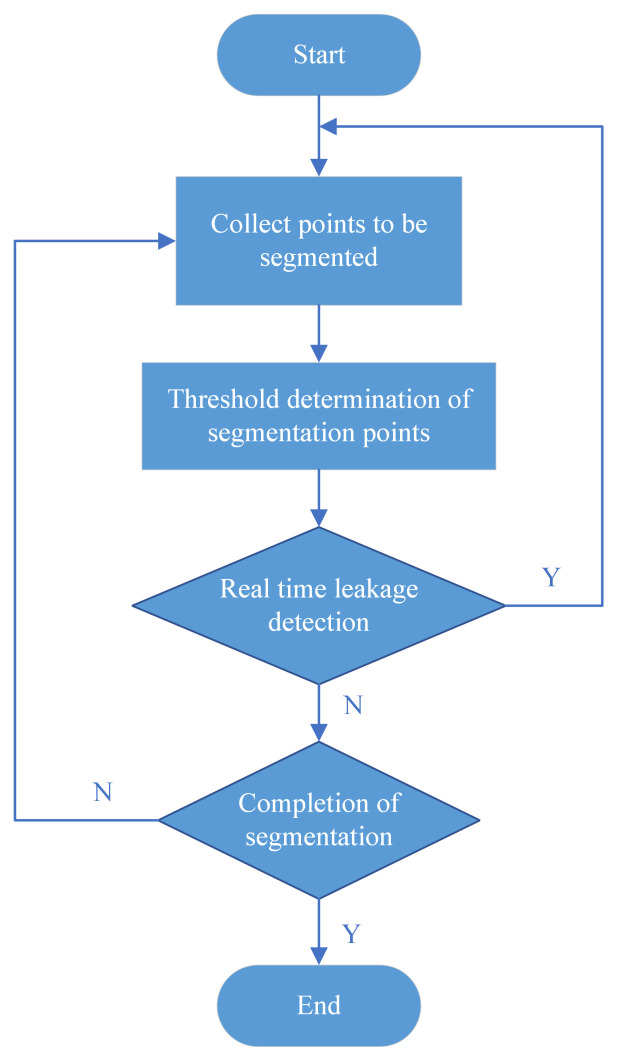
This is the segmentation process within the constraint region, and it does not include parameter adjustments for the constraint region. Through this process, segmentation can be performed from the top to the end of the lung airway tree.

In this paper, the wavefront propagation method is used to extract the points to be segmented, and thresholding is used to determine these points. The segmentation within the region ends when the seed points no longer expand. The specific process is shown in Figure 3. The number of iterations and the result of each iteration can be used for leakage detection. The number of iterations is restricted by the region radius, and according to the general rule of gradual decrease in airway width, the total number of voxels in each iteration result is constrained by the number of elements in the seed point set. Considering special cases such as airway bifurcation and small-range increase in airway width, the parameters of these two restriction methods should be flexible. If leakage is detected, the segmentation of this segment returns to its initial state, and the segmentation threshold decays to maintain segmentation precision, with the decay set to *Threshold* −1. The algorithm for each iteration is shown in Equation (7):

(7) $$X_{\Delta }\left(i,K\right)=f_{\Delta }\left(i,K\right)\left(x,y,z\right)=\mathrm{Imdilate}\left[X\left(i-1,K\right),\mathrm{Sphere}\left(1\right)\right]-X\left(i-1,K\right)$$

where X(i,k) represents the segmentation result after i(i=1,2,3,⋯) iterations of the *K*th seed point set, with $X\left(0,K\right)=S_{\mathrm{area}0}\left(K\right)$. Threshold judgment is applied to $X_{\Delta }\left(i,K\right)$, the criteria of which are shown in Equation (8):

(8) $$f_{\mathrm{region}}\left(K\right)\left(x,y,z\right)\cdot f_{\Delta }\left(i,K\right)\left(x,y,z\right)-Seed\left(K\right)\geq Threshold\left(K\right)$$

If the point to be segmented does not satisfy the threshold condition, its value $f_{\Delta }\left(i,K\right)\left(x,y,z\right)$ is set to 0. Then, the judgment results are superimposed according to Equation (9). The segmentation within the region ends when the iterative results no longer increase.

(9) $$X\left(i+1\right)=X\left(i,K\right)+X_{\Delta }\left(i,K\right)$$

Due to the limitations of thresholds and the influence of noise during segmentation, some voxel points with CT values exceeding the threshold range are erroneously deleted, resulting in the formation of holes, which significantly affects the segmentation results of narrow and small airways. Therefore, before saving the results, it is necessary to refine the segmentation output. In order to preserve the true outer structure of the segmented airways, the algorithm only fills holes inside the airways. Hole filling is achieved by iteratively filling holes within closed regions, which serves as a fundamental operation for morphological operations. Additionally, branching restrictions and region radius adjustments play important roles. Each branching of the bronchial tree alters the segmentation environment, leading to changes in segmentation accuracy and the dynamic range of CT values. In small airways with significant threshold value changes, multiple branchings within the restricted region may result in considerable threshold value differences between the seed point set and the airway voxel points at the region edge, thereby affecting the completeness of segmentation. Therefore, the setting of the radius for region restriction needs to follow the principle of single branching, i.e., the number of branchings within the region should be less than or equal to one.This paper proposes a method for adaptively adjusting the radius of the restriction region. It performs connected component detection on the intersection points between the region boundary and the segmentation, ensuring at most one branching to maintain a consistent segmentation environment for each airway segment from beginning to end to ensure smooth segmentation.

The number of connected components represents the number of airway bifurcations. A bifurcation count of zero means segmentation has not reached the region boundary, so the radius should be reduced to continue segmentation. A count of one means segmentation has reached the region boundary without bifurcation. A count of two means segmentation has reached the region boundary with a single bifurcation. In both cases, the radius is not adjusted. If the bifurcation count is greater than two, it indicates multiple bifurcations, necessitating radius reduction. Radius correction ends when the bifurcation count reaches one or two or when the region radius *R* is reduced to eight. At this point, the segmentation results at the region boundary are extracted, and their area is calculated to determine if leakage has occurred. If no leakage is detected, the segmentation results can be saved. The segmentation results at the region boundary consist of zero or more disconnected small surfaces, which can serve as seed point sets for the next stage of segmentation. The segmentation process ends when no small surfaces remain.

### 3.3. Sealing of Tracheal Wall Gaps

**Figure 4:**
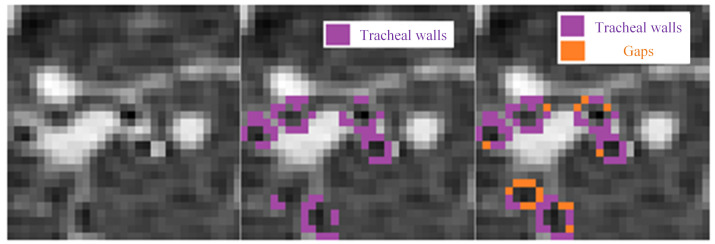
Schematic of tracheal wall gaps. Purple represents the high brightness of the tracheal wall, which can be recognized normally. Orange represents the low-brightness tracheal wall, which is difficult to identify as a tracheal wall gap.

Compared to the low-intensity airway, the bright tracheal wall is a crucial factor in distinguishing the airway from the lung parenchyma. However, due to noise, certain areas of small airways may exhibit insufficient brightness, making these low-intensity tracheal walls prone to leakage, as shown in Figure 4. When segmentation reaches these points, the tracheal wall may fail to prevent the segmentation from spreading from the airway to the surrounding lung parenchyma, resulting in errors. Controlling leakage helps reduce the occurrence of false positives (false detections) and false negatives (missed detections), thereby enhancing overall segmentation performance. This paper refers to these low-intensity tracheal walls as wall gaps, which are a significant cause of frequent leakage in small airways.

**Figure 5:**
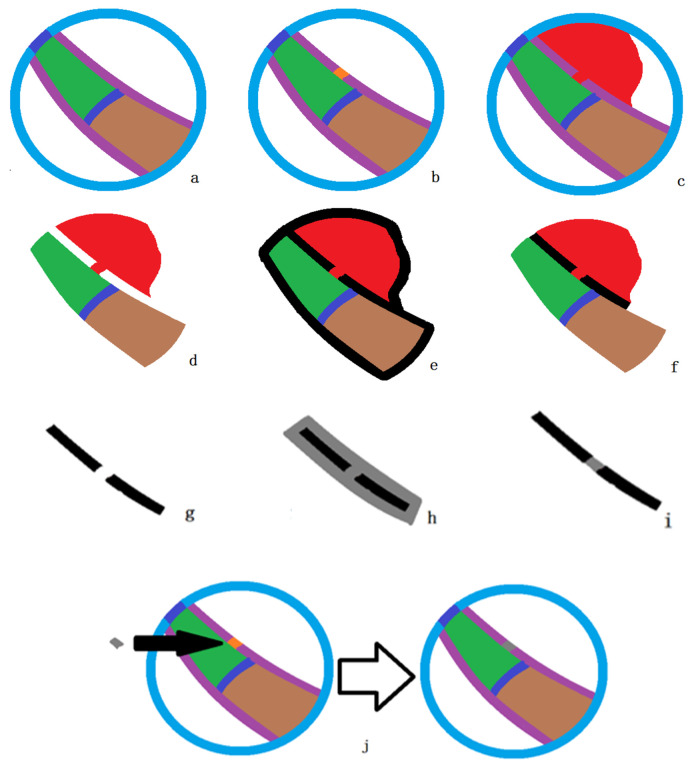
The tracheal wall sealing algorithm model, steps (**a**–**j**), illustrate the specific sealing process. In this model, purple represents the high brightness of the tracheal wall; blue represents the quasi-spherical constrained region; brown represents the previous segmentation region; green indicates the region currently being segmented; dark blue represents the seed point set; red indicates leaks caused by erroneous segmentation; black and gray represent the intermediates generated by morphological operations.

Due to the small and numerous gaps in small tracheal walls, this paper proposes a sealing algorithm for tracheal wall gaps based on quasi-spherical region constraints. This allows the algorithm to detect and seal gaps in the tracheal wall during segmentation, ensuring the smooth progression of lung airway tree segmentation. The model of the algorithm is shown in Figure 5. Figure 5a shows the segmentation result model of a small airway segment. If a gap occurs in the tracheal wall of this segment (as shown in Figure 5b), leakage will occur in the segmentation result (as shown in Figure 5c). At this point, the segmentation result, along with the previous round’s segmentation result and the seed point set of this region, is shown in Figure 5d. This data are denoted as X(K). Figure 5e,f show the dilation and erosion operations performed in sequence, constituting the closing operation on X(K). The dilation operation expands the foreground (usually white voxels) in the image, while the erosion operation, being the opposite of dilation, reduces the foreground (white voxels). The closing operation can fill narrow gaps in the image, which in lung CT scans appear as tracheal wall images. The result after the closing operation, subtracted from the image before the closing operation, gives the exact location of the broken tracheal wall, as shown in Figure 5g. Figure 5h,i also represent closing operations. Subtracting the result in Figure 5i from Figure 5g gives the exact location and size of the tracheal wall gap. Thus, the algorithm specifically increases the CT value at the tracheal wall gaps to seal the low-intensity gaps caused by partial volume effects and noise.

**Figure 6:**
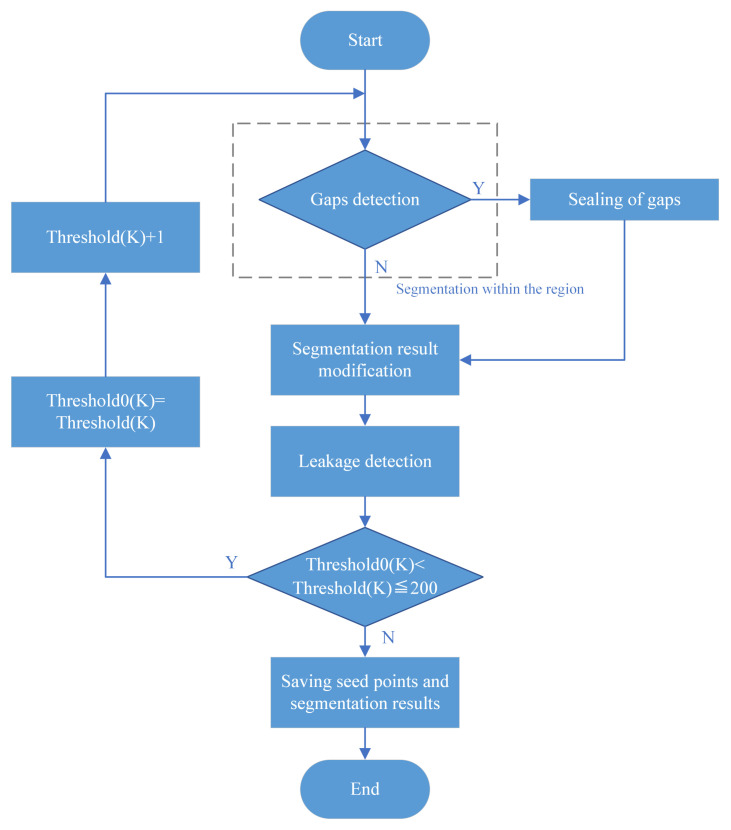
Tracheal wall sealing algorithm flowchart.

To achieve the sealing of multiple tracheal wall gaps and increase the segmentation rate within the airway, the tracheal wall gap sealing algorithm should repeatedly perform detection and execution, as shown in Figure 6. When a tracheal wall gap is detected, the gap is sealed; after recording Threshold0(K) and Threshold(K)+1, the segmentation process is repeated. At this time, the segmentation result should include both correct segmentation and leakage. Continue sealing the tracheal wall gap, as well as increasing the CT value of the voxels at the gap location and the segmentation threshold within the region. Through multiple repeated segmentation processes, the segmentation result obtained after the tracheal wall gap has been sealed, and the threshold increased eliminates the leakage caused by the gap and improves the completeness of the segmentation. The CT difference between the normal tracheal wall and the airway at small airways is about 200, so when the threshold exceeds 200, the gap can be considered completely sealed; the program will exit the loop, and the segmentation result will be saved.

## 4. Results

The EXACT09 Challenge compiled extensive chest CT scan data from different regions using various scanners, scanning protocols, and reconstruction parameters. It selected standardized training datasets (CASE 01–20) and testing datasets (CASE 21–40) based on principles including radiation dose (clinical to ultra-low dose), subjects (healthy volunteers to severe lung disease patients), and acquisition states (full inspiration to full expiration). Experts in chest CT imaging manually annotated these datasets to provide reference standards for lung airway tree segmentation, which were subsequently used to calculate objective metrics for algorithms and establish a unified standard for comparison and analysis [19]. Researchers uploaded their algorithm segmentation results for the website’s datasets, and the site administrators performed segmentation accuracy assessments and parameter validations. The judgment and validation process has not been not publicly disclosed.

The test classifies algorithms into two types: fully automatic and semi-automatic. The results include seven parameters: (1) Branch Count: the number of correctly detected branches. A branch is considered detected if its centerline length is greater than 1 mm. (2) Branch Detected (%): the percentage of detected branches relative to the total number of branches in the reference standard. (3) Tree Length (cm): the total length of the centerlines of all correctly detected branches. (4) Tree Length Detected (%): the percentage of the total tree length in the correctly segmented results relative to the total tree length in the reference standard. (5) Leakage Count: the number of connected regions of “correct” segmentation adjacent to “incorrect” segmentation regions. (6) Leakage Volume (mm^3^): the volume of detected incorrect segmentation regions (total voxel count). (7) False Positive Rate (%): the ratio of the leakage volume to the total volume of all correctly detected regions (total voxel count).

In 2012, Lo et al. [20] completed a comparison and validation of 15 algorithms for segmentation on the EXACT09 dataset. The EXACT09 Challenge website continually updates algorithm comparison results, facilitating lung airway tree segmentation research [14,27,31,32,33,34,35,36]. Since the fixed data representation method for algorithm performance enables the clear recognition and improvement of existing algorithm defects by others and establishing a complete reference method from scratch involves subjective bias and manual operations, forming a comprehensive comparison system is essential for research in this field. Therefore, in recent years, algorithm validation using the EXACT09 dataset has become an important standard for testing lung airway tree segmentation algorithms.

The algorithm presented in this paper was implemented in MATLAB2017a and validated using the test set provided by the EXACT09 website. The results are shown in Table 1. The validation results encompass seven parameters, with the first four indicating segmentation completeness. The higher the value of these parameters, the better the algorithm. The last three parameters indicate segmentation error rates. The lower the value of these parameters, the better the algorithm.

**Figure 7:**
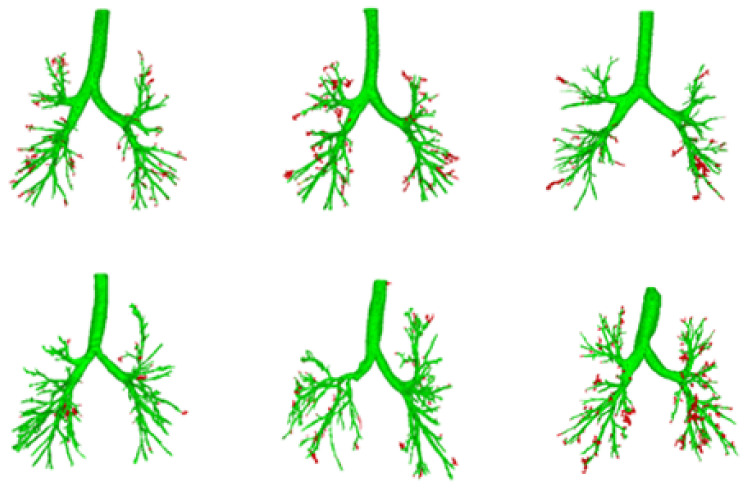
Correct and incorrect markings of some segmentation results. The green part represents the accurately segmented part; the red part represents the incorrectly segmented area.

Some of the segmentation results verified and annotated by the EXACT09 website are shown in Figure 7 in order: CASE22, CASE23, CASE35, CASE36, CASE39, and CASE40. The green images represent the correct segmentation results, while the red dots indicate incorrect segmentation points.

Comparing the mean values of the evaluation parameters for our algorithm, as reported by the EXACT09 website, with 21 other algorithms listed on the website, we found the five segmentation algorithms presented in Table 2. Our algorithm ranked third in all four parameters indicating segmentation completeness, placing it second only to the NagoyaLoopers and FF_ITC algorithms, and showed a noticeable advantage over other algorithms. Most of the algorithms published on the website are based on the region-growing method, with some utilizing morphological and machine learning methods to achieve a certain degree of precise segmentation. However, they did not excel in controlling and handling leakage, which hampered further improvement in segmentation accuracy. It is worth noting that FF_ITC [33] achieved the highest accuracy in segmentation using machine learning. However, it has significant drawbacks: (1) All training samples require manual annotation of both the airway contours and centerlines, which is highly labor-intensive. (2) The small size of the training sample set makes the model prone to overfitting, resulting in poor generalization ability. For the second-ranked NagoyaLoopers [34] algorithm, the rectangular constraint region achieved significant segmentation results. However, the author of this method also explicitly pointed out that the algorithm will cause a large number of leaks. The centerline guided method in WEB2 [35] also achieved good segmentation completion and leak prevention effects. However, it was not as effective as the method proposed in this paper. Experimental results demonstrate that the proposed quasi-spherical region constraint and tracheal wall gap sealing algorithms effectively promoted the segmentation process and significantly improved the basic wavefront propagation method, providing a reliable basis for further research on wavefront propagation methods.

Reducing leakage results in more accurate and reliable segmentation, which can more precisely depict the anatomical structure of the airway tree. This aids doctors in more accurately identifying and assessing pulmonary diseases such as chronic obstructive pulmonary disease (COPD), asthma, and lung cancer, thereby enabling more precise diagnoses. As shown in Table 2, higher segmentation completeness tends to correspond to a higher error rate. Our algorithm performed well in limiting leakage spread, effectively controlling it despite being less favorable at eliminating leakage initiation points, which resulted in a higher Leakage Volume (mm^3^) parameter compared to the top-two algorithms. However, the Leakage Count parameter was not exceptional, indicating some deficiencies in real-time leakage determination and the elimination of initiation points. Improving the ability to eliminate leakage initiation points will significantly enhance the immediacy and reliability of the segmentation results. The False Positive Rate (%) was also leading among most algorithms due to the strong leakage control capability of our algorithm. These three parameters indicate that the algorithm effectively controls leakage spread, even when there are many leakage initiation points; however, there is some deficiency in eliminating these initiation points. This suggests that the quasi-spherical region constraint and tracheal wall gap sealing effectively eliminate leakage, albeit with a noticeable delay. Improving the real-time performance and response speed of these methods can further enhance the algorithm’s immediate reaction ability and reduce leakage occurrence.

Segmentation time, an important parameter indicating the applicability of the algorithm, was also considered. EXACT09 recorded the runtime of 15 algorithms, and Table 3 shows the comparison of computer configurations and runtimes for each algorithm.

**Figure 8:**
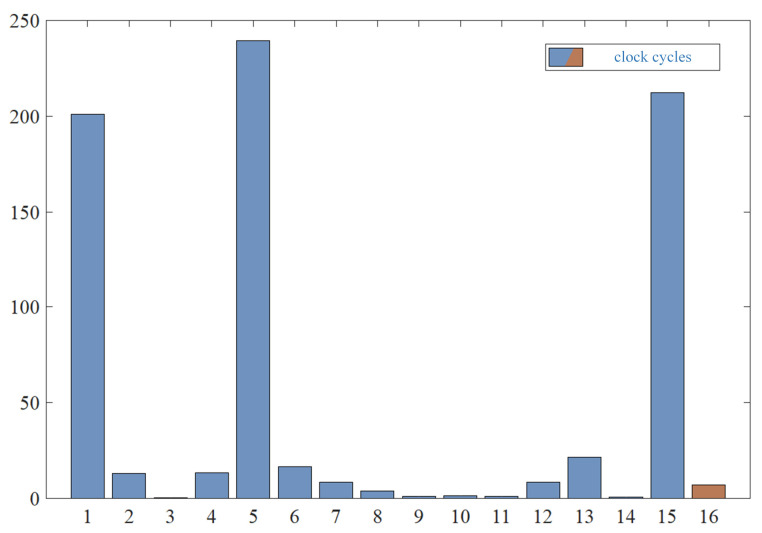
Comparison of algorithm time consumption.

Multiplying the processor frequency by time yields the number of clock cycles the processor spends executing tasks. For algorithms where the processor frequency was not provided, the average value of 2.64 GHz was used. The number of cycles is depicted in Figure 8, with dark blue bars representing the 15 aforementioned algorithms and brown bars representing our algorithm. Table 3 and Figure 8 demonstrate that our algorithm has a significant advantage in terms of time consumption, meeting the real-time requirements for practical applications.

## 5. Discussion

A new algorithm and strategy for accurate segmentation of the pulmonary airway tree have been proposed, which, through validation, have been shown to outperform the top-two algorithms in reducing airway leakage, despite ranking third in segmentation integrity. The results indicate that the algorithm effectively reduces the scope of erroneous segmentation within complex airway structures. This is crucial for improving the overall accuracy of airway tree segmentation, as it can prevent non-airway tissues from being misidentified as airways, thereby enhancing the reliability of clinical diagnosis. Furthermore, the algorithm performed well in leak control, with a low false detection rate. This reduction in false positives can decrease unnecessary interventions and treatments in clinical practice, thereby reducing potential risks to patients. The practical implications of this improvement are significant, as more accurate airway segmentation can lead to better diagnosis and treatment planning for respiratory diseases, ultimately improving patient outcomes.

Compared to existing research, our method introduces new elements. Although similar methods, such as segmentation based on restricted regions and morphological operations, have been explored in previous studies, the innovation of our algorithm provides better performance. Unlike traditional rectangular or cylindrical shapes, the quasi-spherical shape does not require directional discrimination, allowing it to handle the complex branching of pulmonary airways. This improves segmentation accuracy while reducing computation time (as shown in Table 3). Especially in terms of handling and controlling leaks, our tracheal wall gap sealing algorithm ensures good segmentation accuracy while effectively controlling leaks. This comprehensive capability makes it more practical for real-world applications. While the results are promising, the algorithm has limitations in terms of CT imaging quality and the complexity of lung anatomy, especially in cases where airway width undergoes sudden changes. Due to pathological changes in the human body, a small segment of the airway may become extremely narrow, leading to errors in identifying this position as an airway wall defect and executing sealing and causing the algorithm to prematurely terminate segmentation at this point.

Looking ahead, future research may focus on addressing these limitations and further improving algorithm performance. Advanced image processing techniques, such as those based on deep learning methods, are expected to improve segmentation accuracy. Additionally, integrating multimodal imaging data can provide a comprehensive assessment of pulmonary pathology and help refine segmentation algorithms for clinical use. This approach could further enhance the accuracy and reliability of airway segmentation, leading to better clinical outcomes and improved patient care.

## 6. Conclusions

The method proposed in this paper has achieved good results in various indicators under the EXACT09 standardization test, but there are still some shortcomings. Firstly, the algorithm has a lag in limiting leaks, i.e., the segmentation process may retain some leak initiation points due to detection lag, which lowers the overall evaluation of the algorithm. Moreover, the tracheal wall sealing algorithm in this paper is not applicable to all types of sealing. If the gap is too large (exceeding three voxel distances), the effect of the gap sealing algorithm is minimal, and leakage can only be avoided by adaptively adjusting the threshold. This is prone to occur in small terminal airways or post-operative tracheal incisions. Additionally, if the airway undergoes narrow mutations, the algorithm may remove some correctly segmented points during application.

Future work can be carried out in the following aspects. Firstly, we can increase the proportion of real-time leak detection and design parameter adaptive methods for this detection process to enhance the algorithm’s ability to distinguish between segmentation correctness. Moreover, we can improve the algorithm’s detection and smoothing capabilities for airway edges to enhance the overall integrity of the pulmonary airway tree and the accuracy of measuring airway widths, providing a good foundation for virtual bronchoscopy applications in clinical practice. Futhermore, through further analysis of the CT features of peripheral airways connected to alveoli, we can explore methods to achieve the highest possible segmentation goals for the pulmonary airway tree.

By addressing these areas, future improvements can further enhance the clinical utility of our algorithm, making it a valuable tool for respiratory disease diagnosis and treatment planning that ultimately benefits patient care and outcomes.

## Figures and Tables

**Table 1:** Comparasion of test set validation parameters.

Test Data	Branch Count	Branch Detected (%)	Tree Length (cm)	Tree Length Detected (%)	Leakage Count	Leakage Volume (mm^3^)	False Positive Rate(%)
CASE21	122	61.3	64.4	58.3	23	110.5	1.42
CASE22	257	66.4	196.0	59.3	167	1098.7	4.92
CASE23	228	80.3	166.5	64.0	180	1343.3	6.84
CASE24	133	71.5	99.2	61.0	39	922.4	4.38
CASE25	178	76.1	158.9	63.0	53	571.7	2.40
CASE26	39	48.8	28.4	43.3	4	28.9	0.84
CASE27	62	61.4	45.2	55.8	12	110.8	1.86
CASE28	95	77.2	67.3	61.4	9	589.8	6.51
CASE29	134	72.8	90.8	65.7	36	455.8	4.31
CASE30	113	57.9	77.3	50.6	9	159.9	2.07
CASE31	174	81.3	122.0	69.5	47	3220.2	17.42
CASE32	162	69.5	141.0	64.7	53	2324.8	11.19
CASE33	126	75.0	102.8	69.9	49	238.5	2.89
CASE34	288	62.9	214.0	59.9	182	2148.3	8.66
CASE35	274	79.7	224.0	72.4	120	1686.1	7.38
CASE36	258	70.9	247.8	60.1	67	1557.8	6.59
CASE37	141	76.2	121.8	68.5	59	644.8	3.70
CASE38	56	57.1	39.9	60.1	29	922.5	11.38
CASE39	297	57.1	230.9	56.4	117	952.4	4.60
CASE40	307	78.9	279.9	72.3	202	3667.5	12.02

**Table 2:** Algorithm performance comparison.

Test Data	Method	Branch Count	Branch Detected (%)	Tree Length (cm)	Tree Length Detected (%)	Leakage Count	Leakage Volume (mm^3^)	False Positive Rate (%)
FF_ITC [33]	Machine Learning	198.3	79.6	177.1	79.9	115.5	2119.6	11.92
Nagoya Loopers [34]	Region Growing	186.8	76.5	158.7	73.3	35.5	5138.2	15.56
WEB2 [35]	Adaptive Thresholds	161.4	67.2	115.4	57.0	44.1	1873.4	7.27
HybAir [9]	Markov Random Walk	123.8	51.1	91.1	43.9	9.1	351.2	6.78
UAVisionLab [36]	Region Growing	74.2	32.1	51.9	26.9	4.2	430.4	3.63
**Ours**	Wavefront Propagation	172.2	69.1	135.9	61.8	72.8	1137.8	6.07

**Table 3:** Algorithm performance comparison.

Algorithm	CPU/GHZ	Time/min	Clock Cycles/Times
CADTB	2.83	71	200.93
ARTEMIS-TMSP	– –	5	13.11
UAVisionLab	2.4	0.13	0.31
NagoyaLoopers	2.66	5	13.3
DIKU	2.66	90	239.4
VOLCED	1.66	10	16.6
TubeLink	– –	3	8.4
Sevilla	2	2	4
PRLH	3	0.32	1.0
VIA	3	0.5	1.5
ICCAS-VCM	2.4	0.42	1.0
yactaTreeTracer	2.83	3	8.5
GVFTubeSeg	– –	6	21.6
WEB2	– –	0.17	0.6
Iowa-1	– –	59	212.4
**Ours**	3.6	2	7.2

## Data Availability

The raw data supporting the conclusions of this article may be provided upon reasonable requests for scientific research purposes.
